# Nonviral Methods for Inducing Pluripotency to Cells

**DOI:** 10.1155/2013/705902

**Published:** 2013-06-11

**Authors:** Ryan O'Doherty, Udo Greiser, Wenxin Wang

**Affiliations:** Network of Excellence for Functional Biomaterials (NFB), National University of Ireland, Galway, IDA Business Park, Dangan, Galway, Ireland

## Abstract

The concept of inducing pluripotency to adult somatic cells by introducing reprogramming factors to them is one that has recently emerged, gained widespread acclaim and garnered much attention among the scientific community. The idea that cells can be reprogrammed, and are not unidirectionally defined opens many avenues for study. With their clear potential for use in the clinic, these reprogrammed cells stand to have a huge impact in regenerative medicine. This realization did not occur overnight but is, however, the product of many decades worth of advancements in researching this area. It was a combination of such research that led to the development of induced pluripotent stem cells as we know it today. This review delivers a brief insight in to the roots of iPS research and focuses on succinctly describing current nonviral methods of inducing pluripotency using plasmid vectors, small molecules and chemicals, and RNAs.

## 1. iPS: A Journey towards iPS Technology

Somatic cell nuclear transfer (SCNT) was originally hailed in the 1950s as an exciting tool that allowed scientists to probe the developmental potential of a cell. Briggs and King [1] describe this method whereby the recipient egg is activated by pricking it with a glass needle and its nucleus can then be removed using Porters technique. Following this, the egg and donor cell are prepared in a dish and with the use of specialized apparatus are drawn into a needle which damages cell membranes without harming or dispersing the contents of the cell. The cell contents are subsequently transferred to the enucleated egg. The conclusions of these experiments lead to the realization that the irreversible genetic changes that were once thought to be imposed on the genome of differentiated cells were not true, but actually they were reversible epigenetic changes. Groups from around the world continued to experiment in the field on cells derived from mammals, in some cases terminally differentiated cells, and achieved great success in demonstrating that the genomes of even fully specialized cells remained genetically totipotent. However, abnormalities in gene expression were observed in many of these “cloned” mammals which suggested that the reprogramming method was flawed [1, 2].

Work carried out on transcription factors used to switch the lineage of cells hugely contributed and influenced the discovery of inducing pluripotency to cells. These experiments involved introducing lineage-associated transcription factors to certain cells. Under normal conditions these transcription factors are involved in driving cell-type-specific genes and suppressing genes that are involved in promoting other lineages. When introduced to heterologous cells, these transcription factors allow the cell fate to be changed. This discovery was first demonstrated in fibroblasts. Myofibers were formed by transducing fibroblast cells with the skeletal muscle factor MyoD using a retroviral vector [3]. There were continued advances in this area of study where cells from different germ layers were shown to be able to cross these barriers, for instance, the work carried by Ieda et al. [4] which demonstrated fibroblasts converting to cardiomyocytes through exposure to cardiac factors Gata4, Mef2c, and Tbx5.

**Figure 1:**
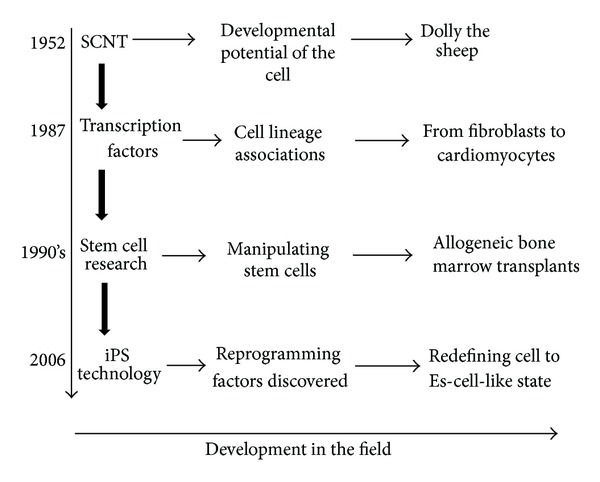
The journey towards iPS.

With past studies proving that cells remain genetically totipotent after differentiation and that it is possible to influence cells to switch between linages, the platform was set for scientists to go a step further and reprogram cells to an embryonic-like state. Although the advent of embryonic stem cell research brought with it many new and exciting techniques that held great promise for the treatment of many diseases, iPS technology supersedes this research in two very important areas. Firstly, iPS cells do not have the same ethical issues surrounding them. This is because there is no use of human embryos as adult cells are being reprogramed. Secondly differentiated iPS cells, which are therapeutically relevant, do not face the same immune rejection following assessment in vitro and after transplantation in genetically identical recipients. This assessment found no evidence of increased amounts of T cells or antigen-specific secondary immune cells [5] (Figure 1).

## 2. iPS: Beginnings

It was Takahashi and Yamanaka's work [6] in 2006 that first pushed forward the subsequent wave of work that is now being carried out on iPS. This seminal work identified a series of transcription factors that when introduced to cells could reprogram them to an embryonic-like state, thus inducing pluripotency to them. An elegant experiment designed to identify factors that could reprogram somatic cells was undertaken. This experiment involved screening a set of 24 pluripotency associated genes that could activate a specific drug resistance allele. After multiple rounds of elimination, Yamanaka and Takahashi were left with 4 specific genes that they believed could reprogram somatic cells. These were Klf4, Sox2, c-Myc, and Oct 4. Upon reprogramming, the resulting iPS cells exhibited various features that are indicative of embryonic stem (ES) cells. These included expression of pluripotency markers such as Nanog: they also generated teratomas in immunocompromised mice when injected subcutaneously and contributed to different tissue development in blastocysts. These results suggested that pluripotency had been achieved, however, further analysis showed that in comparison to true ES cells, levels of pluripotency markers were markedly lower [7]. Together with failing to contribute to the germline and generate chimeras, these iPS cells had a number of issues to overcome before advancements could be made, a challenge that many groups took upon themselves to investigate. The surge in research into applying, improving and reimagining the work Yamanaka and Takahashi first started propelled iPS to the forefront of stem cell research. Rapid advancements were seen in the delivery systems, reprograming factors, and models used to reprogram cells. iPS cells have since been derived from humans [8], rats [9], and rhesus monkeys [10] as well as from different somatic cell populations ranging from keratinocytes [11], neural cells [12], and lymphocytes [13] among others.

## 3. iPS: A Viral Revolution

**Figure 2:**
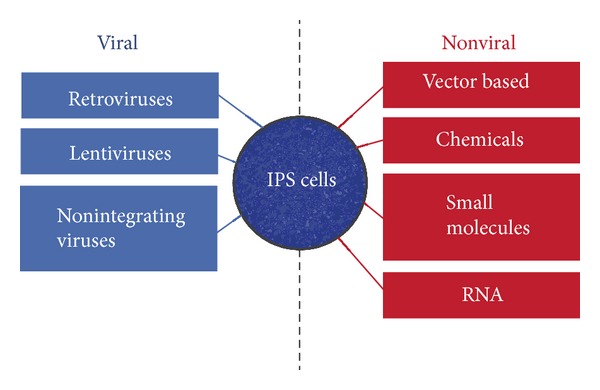
Viral versus nonviral methods for induction of iPS cells.

There are various means of inducing pluripotency to cells, including the use of viral vectors, nonviral vectors, using small molecules accompanied by chemical treatment and finally by RNAs. Each method of iPS has its own advantages and disadvantages. This review will focus on nonviral methods of iPS; however, a brief introduction on viral methods will be given (Figure 2).

There has been a substantial amount of work carried out in the area of virally induced iPS cells which can be subcategorized into three main methods of viral iPS technology. They include the use of retroviruses, lentiviruses, and nonintegrating viruses. Retroviruses were used in producing the first iPS cells which stably integrated into the host genome and introduced the reprograming factors described by Takahashi and Yamanaka [6]. Difficulties arose with this method, however, as the reprogramming remained incomplete due to activation of methyltransferases which meant that the corresponding endogenous genes were not activated [6]. Furthermore, viral transgenes that have been integrated into iPS cells may cause tumor formation in chimeric animals [14]. Although this retroviral reprogramming method gave highly efficient iPS cells, the risk of tumor formation is too great to be applied to a clinical setting and led to other avenues being explored. Studies have taken place to examine if iPS cells can be produced in a way that does not give rise to tumors in chimeric mice which would overcome a great hurdle in viral iPS technology. To this end, Nakagawa et al. generated chimeric mice that survived 100 days using iPS cells that were produced without Myc as a factor. This achievement, however, was accompanied by a reduction in efficiency of iPS generation [15]. Following from this experimental work, Nakagawa et al. continued experimenting with the reprogramming factor Myc in search of reducing tumor formation after reprogramming had been achieved. Results indicated that c-Myc, when used as a factor in reprogramming, was found to increase tumor formation; however, a different Myc family member, L-Myc, was found to promote reprogramming without having tumorigenic repercussions in chimeric mice [16]. An alternate method to avoid tumor formation used by researchers is inducible lentiviral vectors which would allow for the control of expression which is not possible when using retroviruses. This control is exerted by the drug doxycycline which reduces the risk of transgene expression and allows only fully reprogrammed cells to be selected [17]. These inducible vector systems have been used to generate “secondary” methods of reprogramming which do not use direct delivery of reprogramming factors to cells. This is achieved by reprogramming somatic cells using the inducible vector system and then allowing these cells to differentiate in vitro. When this has been achieved, the new somatic iPS cells are cultured in doxycycline containing media and “secondary” iPS cells are formed. These cells represent efficiency levels that are several orders of magnitude greater than the primary iPS cells that were generated [18]. The use of excision strategies has been used to avoid the problem that viral methods have yielded. These excision strategies include the use of the Cre-*lox*P recombination system and piggyBac transposition. By using these systems, undesirable sequences may be removed at a given time allowing for safe reprogramming [19, 20]. There are, however, drawbacks associated with both methods. Firstly, when the Cre-*lox*P method is used, after excision, some vector sequences may be left behind which can cause insertional mutations. Secondly, piggyBac transposition has not been reported in humans and remains a labour intensive process.

## 4. Nonviral/Nonintegrating iPS 

### 4.1. Nonintegrating Vectors

As viral methods of reprogramming showed high efficiency which was desirable, they proved to be too risky to be used in a clinical setting owing to their insertional tendencies. The necessity to find an iPS method that could be used in the clinic was then sought after. Various strategies for nonviral reprogramming have been put forward and will be discussed in the following section.

Okita et al. in 2008 [21] showed that pluripotency could indeed be achieved through nonintegrating viral methods. This was achieved by repeated transfection of two expression plasmids in mouse fibroblasts. One plasmid contained complementary DNA (cDNA) of Oct3/4, Sox2, and Klf4, while the second plasmid contained c-Myc cDNA. This study, although it was carried out on embryonic fibroblasts, demonstrated the ability and potential to reprogram cells in a safe manner. The virus-free iPS cells that were obtained after four rounds of transfection expressed ES marker genes at the same level as ES cells as well as gave rise to chimeric mice, an important standard of pluripotency. Subsequent polymerase chain reaction (PCR) experiments showed no amplification of plasmid DNA in 9 of 11 positive iPS clones implying that there was no integration of the plasmid into the host genome. Southern blot analysis demonstrated that there was no integration of transgenes in the clones. Although a lower efficiency of reprogramming was exhibited, there was proof that virus-free reprogramming could be achieved.

### 4.2. Episomal Vectors

An alternate method of reprogramming cells to an ES-like state is described by Junying et al. in 2009 [22] following the work previously described by Okita. This method of nonviral reprogramming involved using episomal vectors and just a single transfection. In this case, reprogramming was carried out on fibroblasts by transfecting with an episomal vector oriP/EBNA1 (Epstein-Barr nuclear antigen-1) that is derived from the Epstein Barr virus. This vector was chosen as it can be used for transfection without the use of viral packaging and can be removed from cells by a drug selection method. Experimentation on reprogramming efficiency was carried out on several reprogramming factor combinations using lentiviruses. When a higher efficiency was seen, the improved combination of reprogramming factors (OCT4, SOX2, NANOG, LIN28, c-Myc, KLF4, and the SV40 large T gene (SV40LT)) was cloned into the episomal vector oriP/EBNA1, and reprogramming was carried out using IRES2 (an internal ribosome entry site for coexpression) that had been shown to work in experiments using lentivirus vectors. Following the analysis of iPS cell colonies that were found, markers indicative of ES cells were present, as well as similar morphological traits and teratoma formation after injection in immunocompromised mice. As there was no integration into the host genome as confirmed by PCR analysis and due to the loss of cellular episomal vectors in the absence of drug selection, transgene-free iPS cells may be selected through further subcloning. Despite these advantages, this method yields low reprogramming efficiency in human fibroblasts at about three to six iPS colonies per 10^6^ input cells [22]. These frequencies are, however, sufficient to recover iPS cells from a reasonable number of starting cells. 

### 4.3. Minicircle Vectors

In the following year, further advances were made in the field of iPS when Jia et al. [23] published their work on minicircle (MC) vectors that could be used to reprogram human adult cells. They reported that they had constructed a plasmid containing the four reprogramming factors Oct4, Nanog, Lin28, and Sox2 in addition to a green fluorescent protein (GFP) reporter gene. The group were able to excise the bacterial backbone from the plasmid as well as the origin of replication and drug resistance genes by taking advantage of the PhiC31-based intramolecular recombination system which cleaves away the undesired bacterial artifacts and degrades them, leaving MC DNA to be purified containing the desired reprogramming factors. The parental plasmids also contained I-SceI restriction enzyme expression cassettes under the control of an L-arabinose inducible promoter. It was claimed that MC DNA benefited from higher transfection efficiency compared to other plasmids. They also have longer ectopic expression which is due to the lower activation of exogenous silencing mechanisms. In this study, pluripotency was induced to human adipose stem cells. Nucleofection was carried out, and following this, two subsequent rounds were undertaken at days 4 and 6. Analysis after selection and culturing demonstrated a reprogramming efficiency of 0.05% with MC-derived iPS cells. Staining for embryonic markers was positive, and the MC-derived iPS cells exhibited all characteristics associated with pluripotency [23]. This nonviral method of iPS demonstrated a lack of integration into the host genome, an attribute that is desired if the method is to be applicable to a clinical setting, however, reprogramming efficiency still remains lower compared to that of viral methods. The work carried out on iPS MC's gained much support with other groups focusing on this as a means to induce pluripotency. Improvements in MC work was carried out by Chabot et al. [24] who demonstrated the use of electropulsation for MC with GFP delivery to cells. Results showed that there was twofold difference of GFP expression in cells after 3 days between electropulsated MC and parental plasmids. Cellular toxicity was examined, and it was found that the increase of transfection efficiency in MC electropulsated cells was not due to a lack of cellular toxicity as both samples were similarly cytotoxic. In vivo studies also showed increased GFP expression of electropulsated MC, and after day ten, it had expression 36 times higher than that of parental plasmid which could be translated to an iPS scenario [24]. An alternative improvement put forward by Yoshida et al. [25] for iPS cells was to conduct reprogramming in hypoxic conditions. It was found from their study that reprogramming in this condition improved efficiency of reprogramming using both viral and nonviral vectors such as plasmids under 5% O_2_. However, further experimentation needs to be carried out to find the optimal conditions for favourable iPS generation as cytotoxicity remains problematic under such conditions.

Three general approaches are listed previously using nonviral vectors for iPS cell generation. These different methodologies have certain aspects in common; for instance, all three methods avoid integration into the host genome, a considerable achievement, and are carried out in 3 very different ways. Similarly, the three methods that have been attempted generate low reprogramming efficiency, an issue of concern, should the method be used in a clinical setting. Means of enhancing reprogramming efficiency such as culturing in hypoxic conditions and using different methods of transfection are important should iPS be used in a therapeutic approach. Other methods of reprogramming have been studied in a hope to improve upon this drawback in reprogramming efficiency, such as use of small molecules and chemical agents, which will be discussed forthwith (Table 1). 

### 4.4. Small Molecules and Chemical Compounds

The use of small molecules and chemicals is well documented in the literature, and they are used to enhance reprogramming efficiency and iPS cell generation. The idea behind their use is to substitute Yamanaka and Takahashi's original reprogramming factors with a cocktail of chemicals or molecules which will serve to enhance the process. Shi et al. [26] describe how they screened for chemicals and molecules that could do just this. They showed that neural progenitor cells (NPCs), which endogenously express Sox2, were transduced with Oct4 and Klf4 alone (OK) and were successfully reprogrammed to iPSCs. They also showed that this process was greatly enhanced in the presence of a G9a histone methyltransferase inhibitor, BIX-01294 (BIX). Desponts and Ding [27] also carried out work in this area, screening for chemicals and molecules that could be used in conjunction and in place of currently used transcription factors. They claim that an L-channel calcium agonist, BayK8644 (BayK), does not directly cause epigenetic modifications as it works upstream in cell signaling pathways and can therefore avoid unwanted modifications. Other work carried out in this increasingly attractive field includes that of Lee et al. [28] who worked with nanoparticles and iPS generation, Lyssiotis et al. [29] who worked on generating iPS through complementation of Klf4 by chemical means, and Pasha et al. who nonvirally reprogrammed murine myoblasts with a single small molecule, DNA methyltransferase (DNMT) inhibitor, and RG108, to generate cardiac progenitor cells [30]. A prime example of the use of small molecules for replacing transcription factors for reprogramming was discovered by Ichida et al. Their RepSox2 molecule successfully replaces Sox2 by the inhibiting transforming growth factor-*β* (TGF-*β*) signaling, which in turn induces Nanog expression. After screening over 800 compounds, these researchers found that RepSox2 was the only one that could generate iPS cells in the absence of another chemical, valproic acid (VPA). Of important note, when reprogramming using this small molecule, the problematic transcription factor c-Myc was not necessary for inducing pluripotency, and the efficiency of the reprogramming was not compromised [31]. Histone deacetylases (HADC) other than VPA such as suberoylanilide hydroxamic acid (SAHA) and trichostatin A (TSA) also greatly improved reprogramming efficiency [32]. Others carrying out work on small molecules and chemical means for generating iPS cells include Lin et al. [33] who focus on chemical means of treating cells in culture to induce pluripotency [33], Wang et al. [34] who generated iPS cells by retinoic acid receptor gamma and liver receptor homolog 1, and Zhu et al. [35] who induce pluripotency in somatic human cells by OCT4 and chemical compounds. As more groups searched for compounds to replace or indeed enhance the action of known transcription factors for reprogramming, it is interesting to note that Esteban et al., while investigating the role of vitamin C to prevent the build-up of reactive oxygen species in culture after reprogramming, discovered that its presence “alleviates the senescence roadblock during iPSC generation” and increases the performance of reprogramming. This observation was made as there was an increase of proliferation during the middle phase of reprogramming, which the group suggested to be a result of the vitamin C aiding in the bypass of senescence. The authors also postulated that the addition of vitamin c may be influencing reprogramming by interacting with various enzymes [36]. The task of searching for small molecules that can overcome low reprogramming efficiency is being undertaken by many research groups as is highlighted by the work carried out by Zhonghan and Rana on a kinase inhibitor screen for small molecules to aid in reprogramming and iPS generation. A key finding from this study showed that Aurora A kinase negatively affects reprogramming efficiency by inhibiting the inactivation of GSK3*β*. Therefore inhibitors of such molecules would greatly increase iPS generation. Other molecules that were identified as inhibitors of iPS in this study included p38 and inositol trisphosphate 3-kinase [37].

 It can be seen from the vast number of groups working on chemicals and small molecules that there is a strong belief that these methods of reprogramming can efficiently produce genuine, stable iPS cells free of integration and mutation. The use of molecules in reprogramming is seen to be a safe method as they use discrete pathways rather than rely on modification to reprogram cells, which makes this process a safe one. It must be noted, however, that substituting a transcription factor for a chemical compound results in a decreased number of generated iPS clones, which may indicate that a single compound may not be able to entirely replace a functioning transcription factor. 

### 4.5. RNAs

The most recent advancement and developing trend in the field of nonviral iPS work is reprogramming using RNA molecules. Very recently, highly efficient miRNA-mediated reprogramming of mouse and human somatic cells to pluripotency was reported by Anokye-Danso et al. [38] but using integrating viral vectors and not direct transfection of mature miRNAs. A study by Miyoshi et al. [39], however, successfully generated iPS cells by direct transfection of human somatic cells using mature miRNA. Researchers were able to detect GFP expression on day 14 after the transfection of mir-200c, mir-302 s, and mir-369 s family miRNAs, and by day 15, they observed approximately five GFP-positive colonies giving an apparent efficiency that is comparable to that seen with the original report of retrovirus-mediated transcription factor introduction. iPS colonies were generated and passed all standard pluripotency checks. This advancement in the field of iPS technology is exciting due to the lack of any vector meaning no risk of insertional mutagenesis. The use of synthetic RNAs has also been reported, which bypass the innate response to viruses and generate true iPS cells. This work, carried out by Warren et al. 2010. [40], generated iPS cells using this method at high efficiency. BJ fibroblasts transfected with a five-factor modified RNA cocktail (KMOSL) demonstrated iPS cell reprogramming two orders of magnitude higher than those typically reported for virus-based derivations. Moreover, this method far out performed traditional viral methods in regard to the time it took to generate iPS colonies [39]. It is therefore clear that RNA strategies for iPS have come very far in the race to achieve useable iPSCs in the clinic (Table 2).

## 5. Conclusions

From its beginnings in 2006, iPS and its technology have seen many advancements, particularly in the nonviral arena. Attempts to emulate the success of viral reprogramming efficiency, while avoiding the pit fall of integration of undesired DNA into the host genome, have driven this field to where it currently stands. The diverse range of strategies that have been put forward to solve this problem nonvirally demonstrates the commitment and faith the scientific community has in the idea and promise of iPS technology. The applications that may arise from these studies include disease modeling and regenerative tissue engineering which are vitally important contributors to the advancement of medical science. Although this work is yet in its infancy, the awarding of the Nobel Prize for medicine to Yamanaka for his pioneering work in the field, the future of iPS, is certainly bright.

## Figures and Tables

**Table 1:** Advantages and disadvantages of vectors for iPS.

	Advantage	Disadvantage	Efficiency
Nonintegrating vector	Nonintegrating	Low efficiency, need for multiple rounds of transfection	0.001%
Episomal	Nonintegrating, single round of transfection	Low efficiency, labour intensive	0.001%
Minicircle	Nonintegrating, higher transfection efficiency	Potentially cytotoxic	0.005%

**Table 2:** Key literature in the area of vector-based iPS technology, chemical-induced iPS technology, small molecules, and RNA-induced iPS technology.

Vector based	[17, 20–24]
Chemical methods	[31, 33–35]
Small molecules	[26–30]
RNAs	[39, 40]
